# Targeting the Orexin System in the Pharmacological Management of Insomnia and Other Diseases: Suvorexant, Lemborexant, Daridorexant, and Novel Experimental Agents

**DOI:** 10.3390/ijms26178700

**Published:** 2025-09-06

**Authors:** Kacper Żełabowski, Wiktor Petrov, Kacper Wojtysiak, Zuzanna Ratka, Kamil Biedka, Michał Wesołowski, Katarzyna Fus, Dawid Ślebioda, Malwina Rusinek, Maria Sterkowicz, Izabela Radzka, Agnieszka Chłopaś-Konowałek

**Affiliations:** 1 Scientific Society for Psychopharmacology, Department of Forensic Medicine, Wroclaw Medical University, 4 J. Mikulicza-Radeckiego Street, 50345 Wroclaw, Poland; kacper.zelabowski@outlook.com (K.Ż.); wiktor.petrov@student.umw.edu.pl (W.P.); kacper.wojtysiak@student.umw.edu.pl (K.W.); zuzanna.ratka@student.umw.edu.pl (Z.R.); katarzyna.fus@student.umw.edu.pl (K.F.); dawid.slebioda@student.umw.edu.pl (D.Ś.); malwina.rusinek@student.umw.edu.pl (M.R.); maria.sterkowicz@student.umw.edu.pl (M.S.); izabela.radzka@student.umw.edu.pl (I.R.); 2 Department of Physiology and Pathophysiology, Division of Pathophysiology, Wroclaw Medical University, Chalubinskiego 10, 50368 Wroclaw, Poland; kamil.biedka@umw.edu.pl (K.B.); michal.wesolowski@umw.edu.pl (M.W.); 3 Department of Forensic Medicine, Division of Molecular Techniques, Faculty of Medicine, Wroclaw Medical University, Sklodowskiej-Curie 52, 50369 Wroclaw, Poland

**Keywords:** dual orexin receptor antagonists (DORAs), orexin system, hypocretin, suvorexant, lemborexant, daridorexant, insomnia, psychopharmacotherapy, sleep architecture

## Abstract

The orexin (hypocretin) system plays a central role in regulating the sleep–wake cycle through two neuropeptides, orexin-A and orexin-B, which act on OX1R and OX2R receptors. Emerging evidence links heightened orexin signaling with the pathophysiology of chronic insomnia. This review outlines the neurobiology of the orexinergic system, compares the pharmacological profile of dual orexin receptor antagonists (DORAs) to traditional GABAergic hypnotics, and evaluates the clinical efficacy and safety of Suvorexant, Lemborexant, and Daridorexant. DORAs function by selectively dampening orexin-driven arousal, thereby facilitating sleep onset and maintenance without disrupting natural sleep architecture. Clinical trials have shown that these agents significantly reduce sleep latency and enhance sleep continuity, with a favorable side effect profile. Overall, DORAs represent a distinct and clinically advantageous option for insomnia treatment, with growing interest in their potential utility across mood, anxiety, and neurodegenerative disorders.

## 1. Introduction

The orexin (also known as hypocretin) system is critically involved in the neurobiological regulation of the sleep-wake cycle [1]. Orexin-A and orexin-B, two neuropeptides produced predominantly by neurons in the lateral hypothalamus, mediate their physiological effects through two G-protein-coupled receptors including orexin receptor type 1 (OX1R) and type 2 (OX2R) [2]. These receptors are widely expressed across multiple brain regions implicated in arousal and vigilance, including but not limited to the locus coeruleus, dorsal raphe nuclei, thalamus, and cerebral cortex [3]. The distribution and function of the orexinergic network underscore its integral role in maintaining wakefulness and modulating transitions between behavioral states [4]. Dysregulation within this system, especially persistent overactivation of orexin signaling, has been increasingly recognized as a potential contributing factor in chronic insomnia [5]. Consequently, pharmacological agents that inhibit orexin receptors (notably dual orexin receptor antagonists, or DORAs) have emerged as promising alternatives to conventional hypnotics [6].

Sleep fulfills a range of critical physiological and cognitive functions, including cellular restoration, metabolic homeostasis, emotional regulation, and the consolidation of memory [7,8,9,10]. Factors like psychological stress, exposure to noise, artificial light, and disrupted daily routines are key contributors to the development of insomnia. These environmental challenges stimulate the central arousal network, especially the orexin system, thereby interfering with the ability to initiate and maintain sleep [5,11]. Persistent disruptions to sleep, as observed in insomnia disorder, can exert widespread negative effects on both psychological and somatic health [12]. Insomnia is commonly defined by difficulties in initiating or maintaining sleep, or by early morning awakenings, typically accompanied by significant daytime dysfunction [13]. While transient sleep disturbances may remit without intervention, chronic insomnia (defined as symptoms occurring at least three times per week for a minimum duration of three months) frequently necessitates clinical management [14,15]. Moreover, it is seldom an isolated condition, often co-occurring with other psychiatric or medical disorders [16,17].

Epidemiological data indicate that over 80% of individuals diagnosed with major depressive disorder (MDD) report significant disturbances in sleep [18,19]. Importantly, insomnia frequently precedes the onset of depressive episodes and has been identified as a predictive factor for both the initial development and subsequent recurrence of MDD [20]. Furthermore, even following remission of core depressive symptoms, persistent sleep disturbances are strongly correlated with an elevated risk of relapse and reduced therapeutic responsiveness [21]. As such, the effective identification and management of insomnia are essential components in the integrated treatment of mood disorders [22]. Furthermore, sleep disturbances are associated with anxiety disorders, post-traumatic stress disorder (PTSD), and bipolar disorder. In these conditions, hyperarousal, driven by dysregulation of the hypothalamic–pituitary–adrenal (HPA) axis, elevated cortisol levels, and increased autonomic activity, impairs sleep initiation and maintenance [23,24]. These physiological disruptions parallel those observed in chronic insomnia, indicating overlapping neurobiological mechanisms. In PTSD, sleep dysfunction is often compounded by nightmares and circadian misalignment, intensifying main symptoms [25,26]. Accordingly, targeting insomnia may not only relieve a primary complaint but also attenuate broader psychiatric symptomatology [26,27].

Conventional hypnotics, such as benzodiazepines and non-benzodiazepine “Z-drugs” (including Zolpidem), primarily potentiate GABAergic transmission to induce sedation [28]. Although effective acutely, their use is limited by risks of dependence, tolerance, cognitive impairment, and increased incidence of falls and fractures, particularly in the elderly [29,30]. In contrast, DORAs provide a more selective mechanism by attenuating the orexin-mediated arousal pathways implicated in the pathophysiology of insomnia [30,31,32]. By modulating this specific wake-promoting system, DORAs facilitate the initiation and maintenance of sleep without significantly disrupting overall neurophysiological balance [30,32]. This targeted action supports the preservation of normal sleep architecture and promotes sleep patterns that more closely resemble physiological sleep, while minimizing adverse cognitive and sedative effects.

Clinical trials involving agents such as Lemborexant and Daridorexant have demonstrated significant improvements in sleep latency, total sleep duration, and sleep efficiency [33,34]. These compounds also exhibit a favorable safety and tolerability profile, characterized by low abuse liability, minimal withdrawal symptoms, and limited impact on respiratory and cardiovascular function (as backed up by relative absence of such adverse effects in pharmacovigilance data) [35,36,37,38].

Such attributes render DORAs particularly suitable for use in older adults and populations with complex pharmacological regimens [39,40]. At the molecular level, orexin receptor antagonists influence a broad range of neurobiological processes beyond the regulation of arousal [41]. The orexin system plays a modulatory role across several major neurotransmitter circuits, most notably dopaminergic, serotonergic, noradrenergic, and glutamatergic pathways, all of which are commonly dysregulated in mood and anxiety disorders [41,42,43]. Orexinergic projections to the ventral tegmental area and dorsal raphe nucleus facilitate dopamine and serotonin release. Antagonism of orexin receptors may attenuate overactivation within these systems, contributing to mood stabilization and anxiolytic effects [44,45,46]. Moreover, suppression of orexin input to the locus coeruleus may reduce excessive noradrenergic tone, a key factor in the hyperarousal and stress reactivity observed in chronic insomnia [5,31,47]. In cortical regions, orexin signaling enhances glutamatergic excitation, promoting wakefulness. Suppressing this excitatory drive may aid in sleep initiation and consolidation [48,49]. Collectively, these neurochemical effects support the hypothesis that orexin receptor antagonists possess therapeutic potential that extends beyond insomnia, offering adjunctive benefits for comorbid psychiatric symptoms.

This efficacy has been observed in trials extending up to a year in duration [50]. Research revealed that close to 60% of both patients and clinicians reported improvements in their insomnia disorder, with no instances of discontinuation due to treatment-related adverse events. Its low potential for abuse, minimal risk of rebound effects, and lack of impact on cardiovascular or respiratory function make it a suitable option, especially for older adults and those with polypharmacy or a history of substance use [51,52]. For this reason, therapeutic strategies targeting the orexin system represent a promising and innovative approach for the treatment of insomnia.

Despite its widespread occurrence, insomnia remains largely underdiagnosed within current healthcare frameworks [53]. According to estimations, around 9% to 15% of the general population suffers from insomnia, accounting for elevated healthcare expenses and reduced work productivity. Moreover, research has consistently demonstrated a strong association between insomnia and neurodegenerative disorders or vascular diseases, highlighting its significant role in contemporary healthcare challenges [54]. The condition was found to be more prevalent in women, especially those experiencing hormonal changes including menopause, with 1.4-fold higher risk than men. Furthermore, it increases with age, affecting 40–70% of older adults, where frequent nighttime awakenings are the most common complaint. Early morning awakenings tend to be more frequent in those over 65 [55]. Recent studies suggest a significant association between insomnia and an increased risk of myocardial infarction (MI) in both men and women, further substantiating the growing body of evidence that underscores insomnia’s contribution to elevated cardiovascular risk [56]. Additionally, insomnia was found to be a significant factor in major depressive disorder (MDD), contributing to both clinical and economical challenges.

Despite decreasing depression severity, insomnia independently worsens health outcomes, making it a significant contributor in MDD progression [57,58]. Pathophysiology of insomnia involves an elevated state of arousal during non-REM sleep coupled with disturbed circadian rhythms and sleep maintenance. The condition might be categorized as either temporary or chronic based on duration and time intervals between which the symptoms occur. Although, in some cases, the diagnosis could be based purely on the patient’s history, it should be supported by sleep examination, including its duration, habits, and presence of any factors that could potentially affect the sleep quality, including alcohol [59]. The Insomnia Severity Index (ISI) is a seven-item scale used for assessment of both nocturnal and diurnal insomnia symptoms, measuring aspects like sleep onset, maintenance, and its impact on daily functioning, with higher scores reflecting more severe insomnia. In addition to its primary role in sleep medicine, the ISI is also utilized in other fields, including oncology, for examination of the relationship between sleep quality and patient health [60].

## 2. Mechanism of Action of Orexin System

### 2.1. Classical Neurochemistry of Wakefulness

The classical understanding of the neurochemistry of wakefulness primarily revolved around neurotransmitters such as norepinephrine, serotonin, GABA, and histamine. These neurotransmitters, central to early sleep research, were targeted by various pharmaceuticals, ranging from benzodiazepines and Z-drugs to tricyclic antidepressants, which exhibited sedative or stimulant effects. However, the discovery of a novel hypothalamic neuropeptide system added a fundamentally new dimension to our understanding: the orexin (also called hypocretin) system [3].

### 2.2. Orexin Peptides and Receptors

Orexinergic neurons in the lateral hypothalamus produce a precursor polypeptide that is cleaved into two isoforms; orexin A and orexin B. These peptides bind to two G-protein-coupled receptors: orexin receptor 1 (OX1R) and orexin receptor 2 (OX2R) [61]. While both receptors interact with G-protein pathways, their downstream effects differ. OX1R predominantly modulates Na^+^/Ca^2+^ exchange, whereas OX2R enhances NMDA receptor transcription, suggesting a potential influence on synaptic plasticity and, consequently, neurological disease processes [49].

#### 2.2.1. Differential Functions of OX1R and OX2R in Sleep and Arousal Regulation

The orexin receptor subtype OX2R is widely recognized as the principal mediator in the regulation of sleep–wake states [62]. Pharmacological antagonism of OX2R has been shown to reliably promote sleep, forming the basis for the clinical efficacy of DORAs such as Lemborexant and Daridorexant [63]. In contrast, OX1R appears to play a more modulatory role, particularly in relation to affective processes associated with arousal. Emerging evidence suggests that OX1R is implicated in the regulation of anxiety and emotional tension occurring during sleep initiation, rather than in the direct induction of sleep itself [64].

#### 2.2.2. Orexin Receptor Specificity in Reward and Addiction

The orexin system contributes significantly to the regulation of reward-related behaviors, with OX1R playing a predominant role in the reinforcement mechanisms linked to substances such as alcohol, morphine, and cocaine [65]. Evidence indicates that prolonged ethanol exposure, particularly during the developmental window of adolescence, results in heightened orexinergic activity [66]. This hyperactivation is associated with alterations in normal sleep patterns, suggesting a complex interplay between orexin signaling and neurophysiological regulation [67]. Collectively, these findings support the therapeutic potential of orexin receptor modulation, especially targeting OX1R, as an avenue for mitigating addiction and its related behavioral disruptions.

#### 2.2.3. Selective Orexin Receptor Modulation and Therapeutic Progress

The development of selective ligands targeting orexin receptors has opened new avenues for clinical intervention. For instance, TAK-925, a highly selective OX2R agonist, demonstrates promising therapeutic efficacy with a reduced risk of side effects commonly associated with reward system activation [68]. This receptor-specific approach may also prove beneficial in conditions such as binge eating disorder, where OX1R appears to play a more prominent role [69]. Such targeted modulation underscores the potential for refined treatments tailored to distinct orexin receptor functions.

### 2.3. Regional Brain Activity and Orexin Stimulation

Orexin-producing neurons exhibit markedly reduced activity during sleep, with especially low firing rates observed during rapid eye movement (REM) sleep [70]. The functional consequences of orexinergic transmission are closely tied to the differential distribution of orexin receptor subtypes across key arousal-related brain regions. For instance, the locus coeruleus (LC), which is densely populated with OX1R, responds robustly to orexin A and plays a pivotal role in norepinephrine-mediated arousal [71]. In contrast, the tuberomammillary nucleus that is integral to histaminergic neurotransmission primarily expresses OX2R, implicating it in sustained wakefulness [72]. Moreover, several regions involved in dopaminergic and cholinergic signaling, including the ventral tegmental area, basal forebrain, and the pedunculopontine and laterodorsal tegmental nuclei, co-express both OX1R and OX2R. These structures collectively support arousal through diverse, receptor-specific mechanisms, underscoring the complexity of orexin-mediated modulation of wakefulness [73].

#### Inhibition of Sleep-Promoting Centers

Orexin suppresses sleep-promoting areas such as the medullary REM muscle atonia circuits, reinforcing its role in arousal. Serotonin released from the dorsal raphe nuclei acts on 5-HT1A receptors to hyperpolarize orexin-producing neurons, thereby reducing their excitability and contributing to the regulation of sleep onset through a feedback inhibitory mechanism [74,75].

### 2.4. Orexin, Narcolepsy, and Autoimmune Involvement

The orexin system is critically involved in narcolepsy type 1 (NT1), a disorder characterized by excessive daytime sleepiness, cataplexy, and sleep paralysis. A key diagnostic marker is the significant reduction in orexin A in cerebrospinal fluid, reflecting the loss of orexin-producing neurons in the hypothalamus [76,77]. Postmortem studies suggest this neuronal degeneration is driven by autoimmune T-cell activity [78]. NT1 shows a strong genetic association with the HLA-DQB1*06:02 allele, along with specific polymorphisms in T-cell receptor genes, supporting an autoimmune etiology [79]. Beyond sleep regulation, orexins also appear to participate in immune signaling, underscoring their broader physiological relevance [80].

A study of 77 participants explored how sleep deprivation influences inflammation, focusing on white blood cells (WBCs) and granulocytes (GRAs), and the role of physical activity. Sleep loss led to increased granulocyte counts in all individuals, while WBC elevation was mainly seen in those who were more physically active. Poor subjective sleep quality over several days was the strongest predictor of higher WBC levels. Additionally, granulocyte counts were linked to longer REM latency and lower sleep efficiency, highlighting a complex connection between sleep architecture and immune function [81]. Moreover, the research suggests that orexin peptides influence immune responses and neuroinflammation across a range of conditions, including sepsis, inflammatory bowel disease, and neurodegenerative disorders, by downregulating NF-κB activity and promoting anti-inflammatory signaling mechanisms, further highlighting their role in immune signaling [82].

### 2.5. Orexin and the Hypothalamic–Pituitary–Adrenal (HPA) Axis

Orexinergic pathways interact with the hypothalamic–pituitary–adrenal (HPA) axis, modulating levels of ACTH and corticosterone even in the absence of acute stress [83]. This regulatory link may help explain the sleep and mood-related effects of certain traditional Chinese herbal formulations. For example, modified Suanzaoren Decoction appears to stabilize orexin signaling and, in turn, modulate HPA activity, mechanisms thought to underlie its sedative and anxiolytic properties [83,84].

#### Herbal Modulation of Orexin Signaling Pathways

Herbal formulations like Zaoren Granules appear to reduce OX2R expression through serotonin-related mechanisms, affecting appetite, inflammation, and neuroendocrine function, probably via the cAMP/CREB pathway. Chaihu-Longgu-Muli Decoction has shown benefits in kidney disease models by suppressing NF-κB-driven inflammation through orexin-linked pathways. Additionally, orexin under conditions of sleep deprivation may involve signaling through CaMKK2/AMPK and PI3K/Akt/mTOR pathways, reflecting its role in diverse cellular processes.

### 2.6. Orexin Dysregulation Across Neuropsychiatric and Neurodegenerative Disorders

Studies measuring biomarkers have shown that orexin levels vary across different neuropsychiatric and neurodegenerative disorders. In some cases, elevated or decreased levels are associated with cognitive deterioration, but the direction of this relationship heavily depends on the specific condition.

This connection appears to be multifaceted, likely influenced by factors such as the phase of the illness, the body’s adaptive responses, and its interplay with sleep patterns and inflammatory processes. Reduced concentrations of orexin-A in the bloodstream have been linked to impairments in thinking and decision-making abilities among individuals with anorexia nervosa and epilepsy [85]. Lower blood levels of orexin-A in epilepsy were found to be tied to poorer thinking skills and increased inflammation, suggesting possible protective qualities of orexin-A against cognitive decline in this condition [86]. In Alzheimer’s disease, disturbances in the orexin system are closely linked to impaired sleep–wake regulation. Data from animal models and postmortem studies show that increased levels of orexin in the cerebrospinal fluid are associated with disrupted sleep patterns and worsening cognitive function in individuals with Alzheimer’s disease, especially in cases of mild cognitive impairment related to the condition [87,88,89]. Furthermore, a cross-sectional study involving 50 patients with AD demonstrated a significant association between elevated cerebrospinal orexin levels and reduced sleep duration, highlighting a potential role for orexin dysregulation in the progression of AD pathology, thereby confirming previous theories [90]. Evidence suggests that tau accumulation, particularly in regions such as the locus coeruleus, appears to interfere with orexin signaling, thereby exacerbating impairments in sleep regulation and arousal [91,92]. This dysfunction may hold promise as a biomarker or therapeutic target [4]. Likewise, in Niemann–Pick disease type C, degeneration of orexin-producing neurons has been observed, possibly secondary to toxic lipid accumulation, further highlighting the system’s vulnerability in neurodegenerative contexts [82,89,93].

### 2.7. The Influence of Orexins on the Development of Schizophrenia

**Figure 1:**
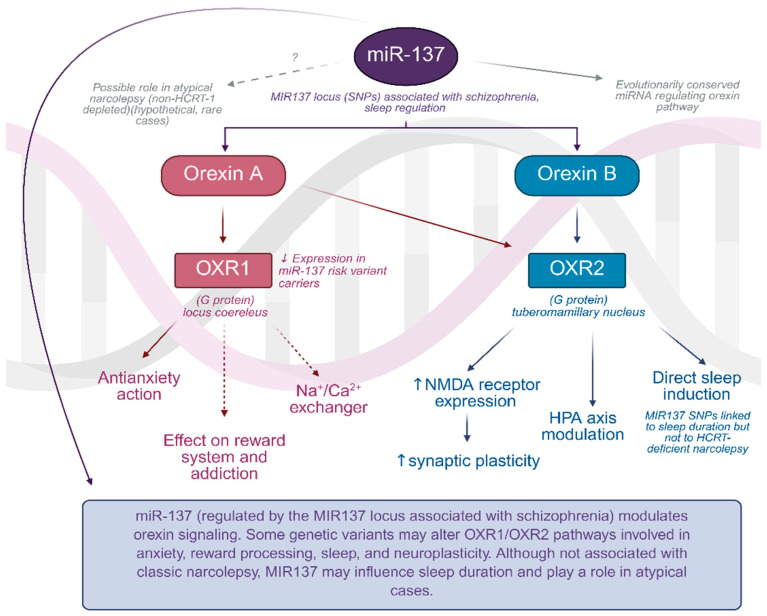
The regulatory role of miR-137 in orexin signaling pathways. Graph was prepared using Biorender. Żełabowski, K.

Interestingly, schizophrenia is not devoid of orexin influence, as the MIR137 locus, which is responsible for silencing miRNA and miR-137, is a marker of genetic predisposition to this disease. Aforementioned miRNA modulates the hypocretin levels and it is conserved across species (Figure 1). The study showed a correlation of the MIR137 locus with sleep length in humans, but unfortunately not with typical narcolepsy cases. Speculation was made, however, that there might be an influence across a specific population of narcolepsy patients in whom HCRT-1 level is not as drastically decreased, but these cases are seldom [94].

### 2.8. Other Disorders Associated with the Orexin System

#### 2.8.1. Anxiety Disorders, Post-Traumatic Stress Disorder, and Bipolar Disorder (BD)

In PTSD and BD, a heightened state of arousal is observed, associated with dysregulation of the hypothalamic–pituitary–adrenal (HPA) axis, elevated cortisol levels, and increased autonomic activity [23,24]. These mechanisms, similar to those described in chronic insomnia, contribute to difficulties in initiating and maintaining sleep. In PTSD, nightmares and circadian misalignment are additional problems that exacerbate core symptoms [25,26].The orexin system, through its impact on arousal, may play a significant role in these processes.

#### 2.8.2. Addictions

The orexin system plays an important role in reward mechanisms, with OX1R being particularly involved in the reinforcement of alcohol, morphine, and cocaine use [65]. Prolonged ethanol exposure, especially during adolescence, has been shown to increase orexinergic activity [66], which is associated with disturbances in normal sleep patterns [67].

#### 2.8.3. Eating Disorders—Binge Eating Disorder

In binge eating disorder, OX1R appears to play a predominant role in regulating feeding behavior. Targeted modulation of this receptor represents a potential therapeutic approach to alleviating symptoms [69].

#### 2.8.4. Epilepsy

By modulating ascending cortical signals and promoting synchronization of cortical firing, the orexin system can reduce discharges typically associated with seizures. This suggests a potential therapeutic role for orexin system modulation in epilepsy management [95].

## 3. Description of Selected Representatives of the Dual Orexin Receptor Antagonists (DORAs) with Side Effects

**Figure 2:**
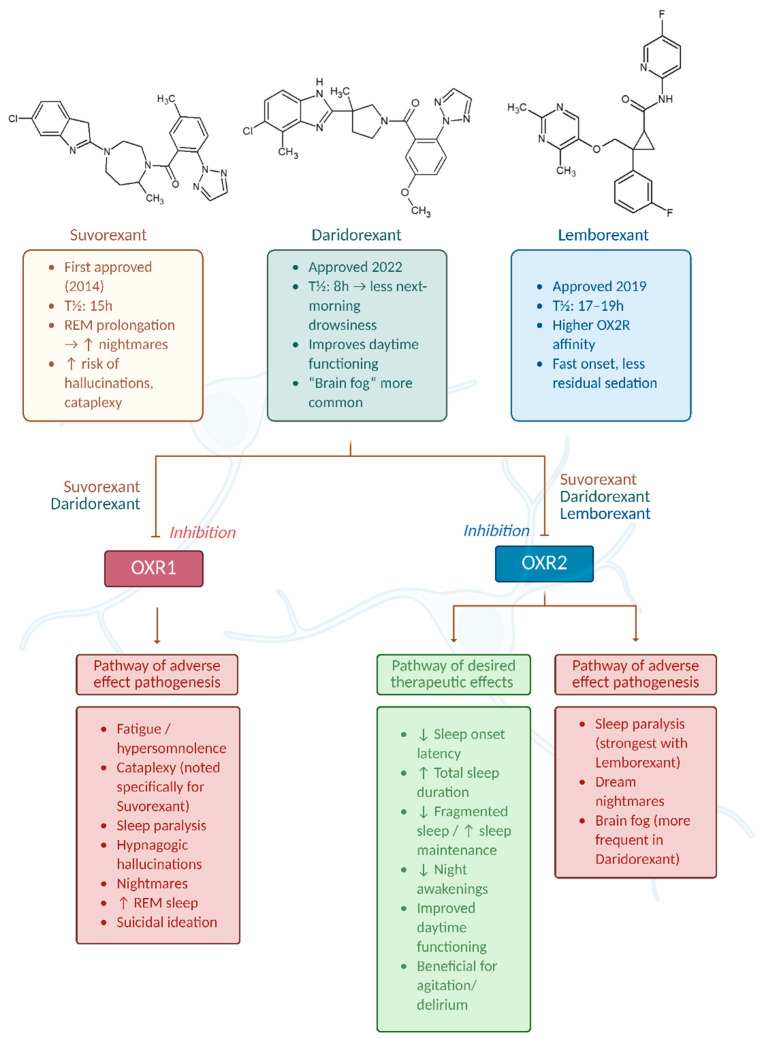
Mechanism of action and differential effects of dual orexin receptor antagonists (DORAs) on OX1R and OX2R pathways. Graph was prepared using Biorender. Żełabowski, K.

The three main agents approved as DORAs by the FDA for insomnia treatment are Suvorexant (Belsomra), Lemborexant (Dayvigo), and Daridorexant (Quiviq) (Figure 2). They work by selective inhibition of orexinergic system signaling in the brain, resulting in quicker sleep onset times and prolonged sleep duration (Figure 2). In this chapter, their characteristics will be expanded upon. Additionally, some other orexinergic agents will be discussed at the end of the chapter, including Seltorexant, which has not been approved yet owing to its unique properties. At the end of the chapter, we will also present Table 1, which summarizes properties of Suvorexant, Lemborexant, and Daridorexant, and Table 2, which contains information about clinical efficacy studies of presented drugs.

We also illustrated the effects of DORA class drugs on OX1R and OX2R receptors in Figure 2.

### 3.1. Suvorexant

#### 3.1.1. Drug Characteristics

As of now, three orexinergic agents have been FDA-approved for pharmacotherapy of insomnia [54], and out of these three, Suvorexant with the trade name Belsomra was the first one to obtain such approval [55], which officially happened in 2014 [58]. Because of its ability to block both OX1R and OX2R orexin receptors, the drug belongs to the dual orexin receptor antagonist class. This specific contribution to regulation of lateral hypothalamic efferent signaling differentiates it from classical hypnotics such as antihistamines or GABA modulators [56].

#### 3.1.2. Pharmacokinetics

For the aspect of pharmacokinetics, the half-life of Suvorexant is approximated to be 15 h, which is the middle ground out of said three agents [56], T_max_ is 2 h, and dosages tested vary between 5 and 20 mg [98]. Its enteral absorption is rapid [98]. Suvorexant’s main metabolic pathway involves CYP3A enzyme isoforms. In consequence, significant drug-to-drug interactions may take place during therapy with Suvorexant and clinically significant differences in Area Under the Curve (AUC) were observed when patients were administered rifampin—a CYP3A inducer—and its inhibitors (ketoconazole, diltiazem). A less significant contribution comes from the CYP2C19 enzyme. The metabolite that is produced by these liver enzymatic reactions is non-active, and excretion of both the metabolite and the parent compound was observed to occur in 66 percent by fecal route and 23 percent in urine. It should be noted that BMI and thus, indirectly, obesity decrease its elimination from the body [58].

#### 3.1.3. Safety and Adverse Effects

Most of the adverse effects of Suvorexant noted in Barriera’s work [96] (based on phase 3 international and multicenter RCT by Michelson et al. lasting 1 year), which showed Suvorexant to be an overall safe agent to use for such an extended period of time, and its tolerance to be high for both elderly and non-elderly patients [96], were not significant, and were characteristic for a plethora of hypnotics, including fatigue, dry mouth, and of course, daytime hypersomnolence [54]. However, some serious reactions also occurred, including sleep paralysis, hypnagogic hallucinations, cataplexy, and suicidal ideation. These findings were attributed to 5% of the participants [54]. Incidence of increased sleep desire in the morning is not as profound though, and Suvorexant is still allowed to be administered to patients with morning arousal disturbances [55]. Asai et al. conducted a safety-oriented study of Belsomra with a large sample of 3428 and found the adverse effect subgroup to include 9.7% of the total sample, with as low as 0.2% constituting drug reactions that were severe, including delirium, agitation, fractures, and subdural hematoma [99,100]. Besides all of that, as Suvorexant does not interfere with benzodiazepine signaling, some traditional and problematic adverse effects typical for past sedatives do not apply to it as much, and findings such as amnesia, confusion, and gait disturbances are not associated with it [58]. For the aspect of the safety of the fetus, as of 2025, there is an absence of research in this area and possible risks have to be evaluated individually. A similar lack of knowledge was mentioned in the aspect of breastfeeding safety with concomitant Suvorexant therapy [58]. A particularly interesting retrospective study regarding the effect of Suvorexant on nightmare occurrence was carried out by Yasuda et al. [59], in which it was shown that Suvorexant prolongs the REM phase of the sleep cycle, which is characteristic for its class, but could also potentially increase the chance of such adverse effects. Said study concluded that the incidence of nightmares was indeed increased, but it was most pronounced in the younger age group, and the elderly seemed to be somewhat resistant to this effect [59].

For the aspect of pharmacovigilance, analysis of 1598 FAERS submissions between 2014 and 2015 made by Ali [101] pointed to a correlation with psychiatric risks and advised prescribers to watch out for changes in behaviors during therapy. Interesting work by Borchert et al. compared FAERS data (years 2015-Q1 through 2017-Q3) with 324 patient self-evaluated drug ratings and complaints found on Drugs.com. Said ratings were shown to be highly similar to FAERS reports in terms of nightmare occurrence, abnormal dreams, sleep paralysis, and other adverse effects [102]. To our knowledge, the most recent pharmacovigilance analysis [38] collected data from Q3rd 2014 to Q4th 2023 and supported earlier findings of nightmares, abnormal dreams, and sleep paralysis being the most common adverse effects.

#### 3.1.4. Clinical Efficacy

Suvorexant’s primary use is for the onset as well as maintenance of sleep [55]. In a study addressing the drug’s therapeutic effect [96] that is mentioned by León-Barriera et al. [54], higher-end doses were used, namely 30 mg for patients above 65 year old and 40 mg for the patients younger than 65 years old, with a notable increase in subjective total sleep length and a decrease in time to fall asleep over the control, placebo group [54]. As insomnia is a chronic condition, it is exceptionally important to address the persistence of the therapeutic effect during such pharmacotherapy. Roughly 62% of patients continued the trial (phase 3 RCT) for 1 year, with therapeutic actions (subjective sleep onset and maintenance improvements) not fading off in such a period of time [96]. Unfortunately, many insomnia comorbidities are contraindications to Suvorexant treatment, including depression, narcolepsy, and obstructive sleep apnea [58]. On top of that, Suvorexant is not considered a go-to alternative for insomnia, as its cost remains relatively high and similarly to Zolpidem, it is associated with notable abuse potential [57]. Aside from its effect on insomnia, Suvorexant was shown to have a positive effect on the extent of delirium associated with Alzheimer’s disease. The underlying mechanism was postulated to involve increased orexin levels in patients with the disease [103].

### 3.2. Lemborexant

#### 3.2.1. Drug Characteristics

The next agent of the DORA class to be brought to the public was Lemborexant. The official FDA approval date was December 2019, and the drug was released the next year by Merck [55]. It is worth noting that Lemborexant has higher in vitro activity for the OX2R receptor isoform compared to Suvorexant [104], and this might contribute to its somewhat more beneficial profile for insomnia treatment, in comparison with Suvorexant [105].

#### 3.2.2. Pharmacokinetics

For 5 mg and 10 mg doses, Landry et al. [106] found the mean half-life to be 17 h and 19 h, respectively [106]. Dosage adjustments are not needed in hepatic dysfunction unless it is moderate; for such cases, 5 mg a night should not be exceeded to avoid excessive adverse effects, particularly daytime sedation [107]. Lemborexant is metabolized by the CYP3A enzyme family in the liver; nevertheless, the influence of kidney injury and insufficiency on the plasma level of the agent was assessed by Landry et al., and was shown to be not worthy of dose adjustment [107]. Concomitant Lemborexant therapy and alcohol exposure is not advised, as alcohol has additive effects on Lemborexant’s plasma level with a subsequent increase in occurrence of negative cognitive symptoms [108]. Shorter time to achieve maximum concentration puts Lemborexant over Suvorexant when quick therapy initiation is needed [104].

#### 3.2.3. Safety and Adverse Effects

In contrast to Suvorexant, Lemborexant was evaluated not to be harmful to patients with obstructive sleep apnea, as a study by Cheng et al. concluded [109]. The abuse potential for the drug is of importance, and it appeared to be comparable to both Zolpidem and Suvorexant [57]. Next morning sleepiness was not associated with the treatment, as evaluated by tests like the Karolinska Scale. Individual features like sex, age, and race had no effect on pharmacokinetics (PK), pharmacodynamics (PD), or overall safety of the therapy [106]. Evaluation of possible emergent adverse effects during long-standing (12 month) therapy was performed by Yardley et al., in an analysis of the SUNRISE-2 study in which no new safety signals were found and Lemborexant was shown to be a safe option for long-term therapy [110].

Considering the relative novelty of Lemborexant and subsequent lack of longitudinal trials, a particularly significant fraction of new safety information can be extracted from pharmacovigilance studies, based on databases such as FAERS. A 2024 study by Jiang et al. [38], also included in Section 3.1, in fact covered both Suvorexant, Lemborexant, and Daridorexant (pan-DORA study). In the same fashion as for Suvorexant, Lemborexant results were in line with previous trials, although it may be worth noting that the sleep paralysis signal was strongest in this agent [38].

#### 3.2.4. Clinical Efficacy

An international 1-month Phase 3 SUNRISE 1 study on the effectiveness of Lemborexant completed by Rosenberg et al., with Zolpidem and placebo as reference points, made it evident that this orexinergic agent was overall the most plausible therapeutic option of the three. Doses tested were 5 mg and 10 mg [60]. SUNRISE 1 successor SUNRISE 2 (Study E2006-G000-303) possesses significant value as it assesses effects for longer, and thus, is more practical in the context of chronic insomnia than 12-month Lemborexant therapy. Therapeutic benefits were persistent in both the first 6 months and total 12 months, as reported by Yardley et al., and a lack of rebound insomnia and withdrawal should also be noted [110]. A small pilot study addressed the effect of Lemborexant on cancer patients with delirium, emphasizing its particular OX2R affinity. The same receptor isoform was shown to have the most influence in agitation states [111]. It should be noted that research of DORAs’ usefulness is highly concentrated on insomnia only. The Kishi group’s network meta-analysis that ultimately covered four studies—SUNRISE-1, SUNRISE-2, and work by Herring et al. [112]—proposed that Lemborexant might be somewhat more beneficial than Suvorexant, but the evidence was not that clear, and data quality was lacking [111]. Later findings by Xue et al. [113] were more promising and showed some superiority of Lemborexant in terms of subjective sleep onset time reduction, subjective total sleep time, as well as subjective wake time after sleep onset [113]; said findings were based on nine RCTs, three of which covered Lemborexant efficacy (SUNRISE 1, 2 and a multicenter study by Murphy et al.) [114]. This study grounded the result of the previous article by Xue’s group.

### 3.3. Daridorexant

#### 3.3.1. Drug Characteristics

Daridorexant is the newest DORA in terms of FDA approval (2022) [54]. In contrast to Lemborexant, Daridorexant exhibits equipotent antagonizing properties to both orexin receptors [115]. It is special in that its short half-life of 8 h could potentially make it a better alternative for patients in whom morning residual somnolence is especially undesired [54]. This feature was the basis for its selection from a plethora of potential pharmaceutical candidates [116].

#### 3.3.2. Pharmacokinetics

Besides its half-life of 8 h, its onset of action is fast, owing to quick absorption [117]. Doses of 25 and the more efficacious [118] 50 mg are officially used [54] in adults at least 18 years old [117]. The hepatic metabolism of the drug is significant with 89% being mediated by CYP3A4, opening doors for many problematic interactions. Additive negative effects of simultaneous Daridorexant and ethanol consumption were noted, same as for Lemborexant [116]. Compromised kidney function, based on C_max_ increase and AUC, did not affect Daridorexant exposure [118].

#### 3.3.3. Safety and Adverse Effects

Residual effects of the therapy, mentioned earlier, were evaluated by Muehlan and Brooks, particularly in the context of their influence on driving performance. A custom-built Green Dino driving simulator was used for this purpose, and its role was to quantify Standard deviation of lateral position (SDLP) as a measure of driving impairment. Zolpidem was used to assess the simulator’s accuracy and it indeed showed a significant decrease in driving performance. On the contrary, Daridorexant was found to have an effect comparable with placebo. However, subjective driving performance was compromised on the initial 2 days of therapy. Thus, individual assessment of potential negative response is recommended [119]. Adverse effects of the Daridorexant regime were similar to other orexinergic agents and were not severe with their occurrence being not dose-dependent [120]. Comorbid psychiatric disorders were not found to change therapy tolerance or safety of Japanese subjects [121]. Daridorexant partook in a particularly interesting study regarding the dampening of responsiveness to external stimuli during sleep [122]. Such an effect is uncompromisingly a significant issue whenever a CNS depressant drug is being used in therapy. Magliocca et al. showed that Daridorexant does not induce such a response, and subsequently, awakening in emergency situations was not disrupted [122]. Even though Daridorexant was observed to favor REM sleep presence, which is known to be involved in dream recalling, it did not induce significant abnormalities in dreaming or the presence of nightmares [56]. A pharmacovigilance study by Jian et al. highlighted ‘’brain fog’’ being more common in patients taking Daridorexant than in those taking the other two agents [38].

#### 3.3.4. Clinical Efficacy

Daytime functioning impairment was not reported during Daridorexant therapy and in fact, for a 50 mg dose, daytime functioning was improved [97]. Similarly to the other two agents, Daridorexant was shown in phase 3 clinical trials to improve sleep quality of patients with insomnia [97,123]. In a recent paper by Cardiroli et al., Daridorexant is mentioned in the context of a possible novel way of managing eating disorders because of their strong association with the orexinergic system [124]. As with previously discussed agents, it is important to assess long-term efficacy and tolerability in light of disease chronicity. Kunz et al. conducted a 12-month extension study with a total of 804 adult and elderly participants. Similarly to earlier orexants, Daridorexant showed to be a safe agent for persistent maintenance therapy [117].

### 3.4. Other Agents

**Figure 3:**
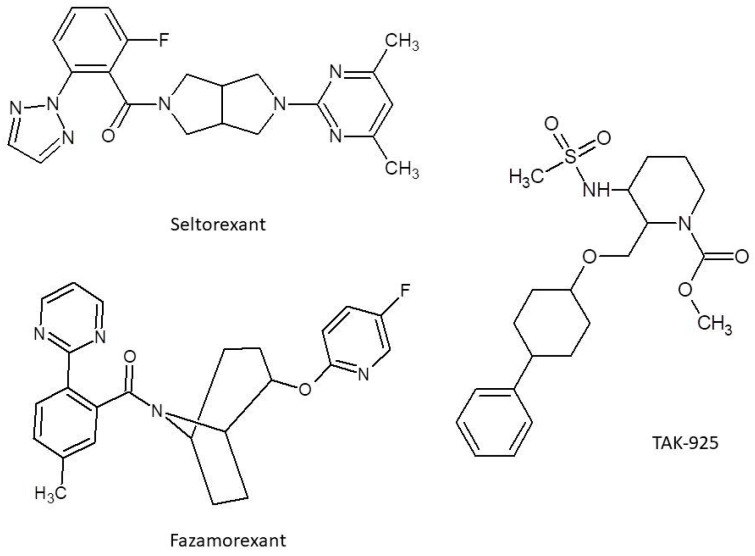
Structure for Seltorexant, Fazamorexant, and TAK-925.

Of note, yet another orexinergic agent currently in a phase III clinical trial, Seltorexant (Figure 3), evoked unexpected antidepressant activity while it was being evaluated for other actions. Such action was observed in monotherapy in MDD patients, but an even more profound outcome was obtained in adjunction with typical antidepressants. Nevertheless, later investigations with broader samples showed no benefit in these conditions and as of now, evidence for it is absent. The particularly short half-life of Seltorexant of 2–3 h is also worth mentioning [125]. TS-142, another experimental agent, was assessed in the context of efficacy in insomnia by Uchiyama et al. [126]. TS-142′s favorable pharmacokinetics (fast absorption and rapid elimination) as well as absence of significant residual side effects and overall positive results of clinical efficacy and consistency in Uchiyama’s study, despite the relatively small sample size, put this agent into the spotlight for the future of orexinergic system-oriented therapy [126]. Yet another agent, Fazamorexant, showed to have promising kinetics as well as safety, as pointed out in Ni’s randomized controlled trial [127]. Fazamorexant’s most common adverse effects came to be drowsiness and dizziness, although the small sample size is also a feature of this study and should be taken into account. Possible saturation of Fazamorexant’s (Figure 3) absorption process should also be considered [127]. Going further, a compound known as TAK-925 (Figure 3) or Danavorexton needs a mention, as it is an agonist of orexinergic receptor 2, contrary to all of the other drugs mentioned in this paper, which are antagonists. Because of this opposite molecular mechanism, it is not being researched in the area of insomnia, but rather in the acceleration of emergence from anesthesia produced by opioids like fentanyl [128].

## 4. DORAs in Contrast to GABAergic Hypnotics: Comparison of Clinical, Economic, and Environmental Perspectives

It is essential to contextualize DORAs alongside GABAergic hypnotics. Benzodiazepines and Z-drugs are known for risks in tolerance, dependence, and falls in the elderly, increasing both health and environmental burdens [129]. Conversely, DORAs present lower potential for overdosing and fewer cognitive or motor side effects, thus positioning them as potentially safer therapeutic options [113].

### 4.1. Impact of DORAs and GABAergic Hypnotics on Sleep Architecture and Neural Pathways

In contrast to conventional hypnotic agents—such as benzodiazepines and non-benzodiazepine “Z-drugs”—which exert their sedative effects by potentiating GABAergic inhibition, orexin receptor antagonists facilitate sleep through a distinct mechanism that preserves physiological sleep architecture [28]. Traditional GABAergic sedatives often alter the natural distribution of sleep stages, potentially diminishing restorative sleep and contributing to residual daytime drowsiness. By selectively targeting the arousal-promoting orexin system, agents such as DORAs maintain the proportional integrity of REM and non-REM sleep, thereby enhancing overall sleep quality with a lower risk of next-day impairment [31,130]. Notably, the ventrolateral preoptic nucleus (VLPO), a key GABAergic node involved in sleep initiation and maintenance, exerts inhibitory control over orexin-producing neurons [131]. This interaction represents a critical point of convergence between GABAergic and orexinergic systems, highlighting the complementary roles these pathways play in sleep–wake regulation.

### 4.2. Question of Healthcare and Economic Impact of DORAs Compared to GABAergic Hypnotics

From the perspective of the healthcare system, although DORAs stand out to have higher acquisition costs than GABAergic agents, their superior safety profile—particularly the reduced risk of falls, dependence, and cognitive impairment [132]—may result in downstream savings and contribute to a more efficient healthcare model.

Moreover, recent cost-effectiveness analyses suggest that Lemborexant may provide favorable value compared to Suvorexant or Zolpidem, supporting its rational integration into healthcare systems [133]. Behavioral interventions such as cognitive behavioral therapy for insomnia (CBT-I) is to remain the most environmentally sustainable approach, lacking pharmaceutical residues and demonstrating cost-effectiveness compared with medication or no treatment [134].

Improved sleep quality itself yields broader health and sustainability advantages, including reduction in comorbidity-related medication use and hospital admissions [135]. Decline in both the number of prescriptions issued and the number of hospital admissions has an effect of reducing both pharmaceutical residues and healthcare-related emissions. This outcome serves to underscore the potential environmental co-benefits of such actions.

### 4.3. Environmental Footprint of GABAergic Hypnotics and Orexin Antagonists

Traditional GABAergic hypnotics such as benzodiazepines and “Z-drugs” are detected in wastewater and surface waters due to their environmental persistence and resistance to standard treatment processes (e.g., diazepam, oxazepam, bromazepam; [136]. In contrast, data on the environmental footprint of dual orexin receptor antagonists (DORAs) are limited, but their generally shorter elimination half-lives and lower risk of bioaccumulation may imply reduced ecological impact, though systematic ecotoxicological assessments are needed.

## 5. Discussion

The introduction of dual orexin receptor antagonists (DORAs) has profoundly altered the pharmacological approach to insomnia treatment, providing a targeted and physiologically coherent solution to the disorder. In contrast to traditional sedative-hypnotic agents, such as benzodiazepines and non-benzodiazepine “Z-drugs,” which broadly potentiate GABAergic inhibition, DORAs act by selectively modulating the orexinergic arousal system, thereby facilitating the onset and maintenance of sleep without disrupting natural sleep architecture [54,55]. This distinction offers a number of clinical benefits, particularly in terms of safety, tolerability, and long-term efficacy.

Suvorexant, the first FDA-approved DORA, has demonstrated efficacy in reducing sleep latency and improving its maintenance [55]. Its dual mechanism, targeting both OX1R and OX2R, makes it effective across a broad spectrum of insomnia presentations. However, its relatively long half-life (~15 h) raises concerns about next-day sedation, especially in vulnerable populations such as the elderly [58]. Although studies report generally mild adverse effects, including fatigue and dry mouth, rare but serious events such as sleep paralysis and hallucinations have also been documented [54]. Notably, Suvorexant has been linked with increased incidence of nightmares, particularly in younger users [59]. Lemborexant, approved in 2019, offers an improved pharmacokinetic profile, with a more favorable receptor selectivity for OX2R, which is predominantly involved in sleep–wake transitions [104]. The receptor specificity of the aforementioned pharmaceutical agent has been demonstrated to result in a number of clinical benefits, including a reduction in residual sedation and a decreased risk of respiratory depression. This characteristic renders the pharmaceutical agent a safer alternative for patients suffering from comorbid conditions, such as obstructive sleep apnea [109]. Comparative studies indicate that Lemborexant may offer superior efficacy in reducing wake time after sleep onset and increasing total sleep duration compared to Suvorexant [113].

Practical prescribing considerations are increasingly important in the clinical use of DORAs. It is noteworthy that in contrast to conventional hypnotics such as Zolpidem (≈20 USD/month), DORAs are substantially more expensive, with average monthly costs of approximately USD 290 for Lemborexant and USD 380 for Suvorexant in the United States [137]. Although manufacturer-sponsored savings programs reduce out-of-pocket costs for many commercially insured patients to ≤USD 35 per month.

Outside of the US, pricing varies considerably, with the UK National Institute for Health and Care Excellence (NICE) reporting a price for Daridorexant of GBP 2.12 per day (≈83.8 USD/month) [138].

A cost-effectiveness model from Japan suggests that when considering both benefits and cost offsets such as reductions in falls and collision, the use of Lemborexant may offer a more favorable balance of “quality-adjusted life-years” (QALYs) compared to Suvorexant or Zolpidem [133].

In the case of Daridorexant, the most recent addition to the DORA class, it stands out due to its shorter half-life (~8 h), which significantly minimizes the risk of next-day sedation [117]. Its balanced affinity for OX1R and OX2R receptors enables it to address both sleep onset and maintenance difficulties. The efficacy of the treatment has been confirmed through a series of clinical trials. These trials have demonstrated that the treatment is effective in improving objective and subjective sleep parameters, without compromising daytime functioning [97]. Importantly, Daridorexant has shown no significant effect on psychomotor performance, as demonstrated in driving simulation studies [119]. This profile renders it an appealing option for individuals who require high levels of daytime alertness.

The orexin system’s broader neurobiological functions, in addition to its role in insomnia, present significant therapeutic prospects. Orexin signaling intersects with key neurotransmitter pathways implicated in mood, stress, and arousal regulation, including the serotonergic, noradrenergic, and dopaminergic systems [42,139]. Suppression of orexinergic tone may attenuate hyperarousal and overactivation of the hypothalamic–pituitary–adrenal (HPA) axis, which are central to both chronic insomnia and comorbid psychiatric disorders such as anxiety, PTSD, and major depressive disorder [20,23].

Clinical and preclinical studies have begun exploring the potential of DORAs in reducing psychiatric symptoms. By dampening orexin input to the locus coeruleus and dorsal raphe nucleus, these agents may help modulate stress reactivity and emotional dysregulation [45]. These findings underscore the potential of orexin antagonists as adjunctive therapies in mood and anxiety disorders, especially when sleep disturbances are prominent.

Another area of growing interest is the role of the orexin system in neurodegenerative diseases. Alterations in orexin signaling have been linked to sleep disturbances in Alzheimer’s disease, with increased orexin levels correlating with disrupted REM sleep and accelerated cognitive decline [87]. Furthermore, tau pathology in brainstem nuclei may interfere with orexinergic regulation, presenting a possible target for future intervention [92].

Despite the considerable promise inherent in DORAs, it is imperative to address the limitations that are associated with this technology. Long-term safety data remain limited, and questions persist regarding their use in special populations such as pregnant or lactating women, patients with hepatic impairment, and individuals with histories of substance abuse [58]. Moreover, while DORAs generally exhibit lower abuse potential than benzodiazepines, they are not devoid of risks, and pharmacovigilance remains essential [38,57].

Future research endeavors should prioritize the delineation of the comparative effectiveness of individual DORAs. Additionally, there is a need to explore their adjunctive use in psychiatric and neurodegenerative disorders. Furthermore, there is a need to refine biomarkers to identify patients that are most likely to benefit from orexin-targeted therapies.

## 6. Conclusions

The usage of dual orexin receptor antagonists (DORAs) signifies a substantial progression in the management of insomnia, as they provide a more precise approach in contrast to conventional sedative-hypnotics, offering targeted efficacy with a favorable safety profile. DORAs have influence over sleep, mood regulation, neuroprotection, and overall quality of life. By selectively modulating the orexin system, DORAs facilitate natural sleep without impairing sleep architecture. The clinical evidence supports their efficacy, safety, and favorable tolerability profiles, particularly with agents like Lemborexant and Daridorexant. Beyond the indication for insomnia, DORAs have managed to demonstrate efficacy in the management of psychiatric and neurodegenerative disorders, attributable to their comprehensive impact on mood and arousal regulation. Nevertheless, the long-term safety data and usage in special populations necessitate further investigation. With ongoing research and refinement, these agents may become cornerstone therapies in both sleep medicine and integrative neuropsychiatric care.

## Figures and Tables

**Table 1:** Properties of Suvorexant, Lemborexant, and Daridorexant.

Drug	Suvorexant (Belsomra)	Lemborexant	Daridorexant
Class of drug	Dual Orexin Receptor Antagonist	Dual Orexin Receptor Antagonist	Dual Orexin Receptor Antagonist
Year of FDA approval	2014	December 2019	January 2022
Half-life	15 h	17–19 h	8 h
T_max_	2 h	1–3 h	1–2 h
Tested dosage	5–20 mg	5–10 mg	25–50 mg
Clinical efficacy	Insomnia Delirium associated with Alzheimer’s disease	Insomnia	Insomnia Eating disorders
Contraindications	depression, narcolepsy and obstructive sleep apnea	Alcoholism	Alcoholism
Adverse effects	asthenia, xerostomia, excessive daytime sleepiness, sleep paralysis, hypnagogic hallucinations, cataplexy, and suicidal ideation	somnolence; daytime functional impairment; sleep paralysis; hypnagogic/hypnopompic hallucinations; cataplexy-like symptoms; parasomnias	cognitive impairment, tachyphrenia, nocturnal sleep-related eating disorder, hypersensitivity, xerostomia, palpitations
Safety to fetus	Absence of research	Absence of research	Absence of research
Breastfeeding safety	Absence of research	Low amounts in milk; child should be monitored	Low amounts in milk; child should be monitored

**Table 2:** Clinical efficacy studies of presented drugs.

Drug	Participants	Tested Dosage of Active Drug	Results	Reference
Suvorexant	Insomnia patients, 18 y.o. or older (470 completed)	40 mg (patients < 65 y.o.) 30 mg (patients ≥ 65 y.o.)	At month 1: Drug: Total sleep time (sTST), min → 40.9 (36.7 to 45.0); Time to sleep onset (sTSO), min → −19.2 (−22.5 to −16.0) Placebo: STST, min → 17.5 (11.7 to 23.4); STSO, min → −9.0 (−13.6 to −4.3)	[96]
Lemborexant	Insomnia participants 55 y.o. or older (962 completed)	5 mg and 10 mg	At day 30: Drug 5 mg: Latency to Persistent Sleep (LPS), min → 24.3 Wake-After-Sleep Onset (WASO), min → 34.5 Drug 10 mg: LPS, min → 17.5 WASO, min → 35.2 Placebo: LPS, min → 32.1 WASO, min → 41.0	[60]
Daridorexant	Adults aged 18 y.o. or older with insomnia	10, 25, and 50 mg	At month 1: Drug 50 mg: LPS, min → −31.2 (−34.5 to −27.9) WASO, min → −29.0 (−32.7 to −25.3) Drug 25 mg: LPS, min → −28.2 (−31.5 to −24.8) WASO, min → −18.4 (−22.1 to −14.7) Placebo: LPS, min → −19.9 (−23.2 to −16.5) WASO, min → −6.2 (−9.9 to −2.5)	[97]

## Abbreviations

The following abbreviations are used in this manuscript:

OX1R	Orexin Receptor Type 1
DORA	Dual Orexin Receptor Antagonist
PTSD	Post-traumatic Stress Disorder
HPA	Hypothalamic–Pituitary–Adrenal
MI	Myocardial infarction
MDD	Major Depressive Disorder
ISI	Insomnia Severity Index
LC	Locus Coeruleus
VLPO	Ventrolateral Preoptic Nucleus
NT-1	Narcolepsy Type 1
BD	Bipolar Disorder
